# Visualization of Oxidative Stress Induced by Experimental Periodontitis in Keap1-Dependent Oxidative Stress Detector-Luciferase Mice

**DOI:** 10.3390/ijms17111907

**Published:** 2016-11-16

**Authors:** Kota Kataoka, Daisuke Ekuni, Takaaki Tomofuji, Koichiro Irie, Muneyoshi Kunitomo, Yoko Uchida, Daiki Fukuhara, Manabu Morita

**Affiliations:** 1 Departments of Preventive Dentistry, Okayama University Graduate School of Medicine, Dentistry and Pharmaceutical Sciences, 2-5-1 Shikata-cho, Kita-ku, Okayama 700-8558, Japan; de18017@s.okayama-u.ac.jp (K.K.); tomofu@md.okayama-u.ac.jp (T.T.); coichiro@md.okayama-u.ac.jp (K.I.); de19013@s.okayama-u.ac.jp (M.K.); de20006@s.okayama-u.ac.jp (Y.U.); de20041@s.okayama-u.ac.jp (D.F.); mmorita@md.okayama-u.ac.jp (M.M.); 2 Advanced Research Center for Oral and Craniofacial Sciences, Okayama University Dental School, 2-5-1 Shikata-cho, Kita-ku, Okayama 700-8558, Japan

**Keywords:** periodontitis, oxidative stress, Nrf2, luciferase activity, heme oxygenase-1

## Abstract

The aim of this study was to investigate whether a Keap1-dependent oxidative stress detector-luciferase (OKD-LUC) mouse model would be useful for the visualization of oxidative stress induced by experimental periodontitis. A ligature was placed around the mandibular first molars for seven days to induce periodontitis. Luciferase activity was measured with an intraperitoneal injection of d-luciferin on days 0, 1, and 7. The luciferase activity in the periodontitis group was significantly greater than that in the control group at seven days. The expressions of heme oxygenase-1 (HO-1) and malondialdehyde in periodontal tissue were significantly higher in the periodontitis group than in the control group. Immunofluorescent analysis confirmed that the nuclear translocation of nuclear factor erythroid 2-related factor 2 (Nrf2) occurred more frequently in the periodontitis group than in the control group. This study found that under oxidative stress induced by experimental periodontitis, the Nrf2/antioxidant defense pathway was activated and could be visualized from the luciferase activity in the OKD-LUC model. Thus, the OKD-LUC mouse model may be useful for exploring the mechanism underlying the relationship between the Nrf2/antioxidant defense pathway and periodontitis by enabling the visualization of oxidative stress over time.

## 1. Introduction

Periodontitis is a chronic inflammatory disease of the periodontal tissue that is caused by the accumulation of dental plaque biofilm and its products on the tooth surface [1]. In the disease process, host cells, including polymorphonuclear leucocytes, produce reactive oxygen species (ROS) as part of the host defense against bacterial pathogens [2]. However, excessive ROS production beyond antioxidant defenses induces damage to DNA, proteins, and lipids in host tissue (oxidative stress) [3]. Experimental periodontitis models have shown that oxidative stress is involved in the progression of periodontitis [4,5,6,7].

**Figure 1:**
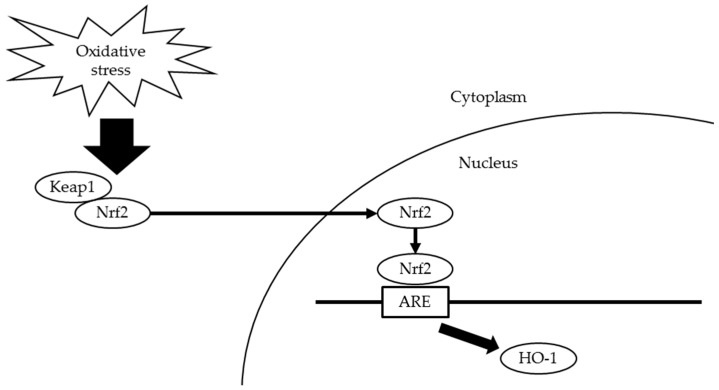
Scheme for Keap1-Nrf2 defense pathway. Under oxidative stress, the Keap1-Nrf2 complex dissociates and Nrf2 translocates into nuclei to bind AREs. Nrf2 activates HO-1 to attenuate cellular oxidative stress. Keap1, Kelch-like ECH-associated protein 1; Nrf2, nuclear factor erythroid 2-related factor 2; AREs, antioxidant response elements; HO-1, heme oxygenase-1.

In orchestrating cellular antioxidant defenses, the redox-sensitive transcription factor nuclear factor erythroid 2-related factor 2 (Nrf2) plays a key role [8,9]. Under normal conditions, Nrf2 interacts with Kelch-like ECH-associated protein 1 (Keap1) that limits Nrf2-mediated gene expression [10,11]. However, under oxidative stress, the Keap1-Nrf2 complex dissociates and Nrf2 translocates into nuclei to bind antioxidant response elements (AREs) [12]. Nrf2 activates antioxidant defense enzymes, including heme oxygenase-1 (HO-1), to attenuate cellular oxidative stress [13] (Figure 1). Some studies have shown that the activation of Nrf2 is involved in oxidative stress due to periodontitis [7,14].

Recently, a Keap1-dependent oxidative stress detector-luciferase (OKD-LUC) mouse model has been developed to visualize Nrf2 expression, and it allows real-time monitoring of oxidative stress [15]. Activation of the Keap1-Nrf2 pathway has previously been monitored using Western blotting or Northern blotting after the animals were killed, but these require cell lysis and involve multiple complicated procedures, and are time-consuming [15]. In contrast, the OKD-LUC mouse model uses a luminescence reaction to overcome these problems, providing a simple, less invasive, and highly sensitive in vivo detection method [15]. Despite the advantages of the OKD-LUC mouse model, little is known about its usefulness in periodontology. We hypothesized that the OKD-LUC mouse model would be useful for exploring the mechanism underlying the relationship between the Nrf2/antioxidant defense pathway and periodontitis. The aim of this study was to investigate whether the OKD-LUC mouse model could be used to visualize the oxidative stress induced by experimental periodontitis. We showed that experimental periodontitis induced oxidative stress (i.e., an increase in malondialdehyde (MDA)) and luciferase activity, indicating that Nrf2 translocated into the nucleus and increased the levels of the antioxidant defense enzyme HO-1.

## 2. Results

**Figure 2:**
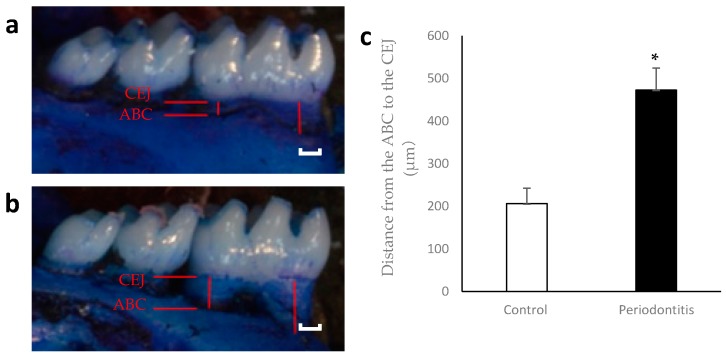
Alveolar bone loss at day 7. Representative photographs of mandibular bone in the control (**a**) and periodontitis (**b**) groups are shown. Red lines indicate the distance from the alveolar bone crest (ABC) to the cemento-enamel junction (CEJ). The distance was significantly higher in the periodontitis group than in the control group at day 7 (**c**) (* *p* < 0.05, *t*-test) (*n* = 6/group). White scale bar = 200 µm.

There were significant differences in the distance from the alveolar bone crest (ABC) to the cemento-enamel junction (CEJ) between the periodontitis and control groups at day 7 (Figure 2).

**Figure 3:**
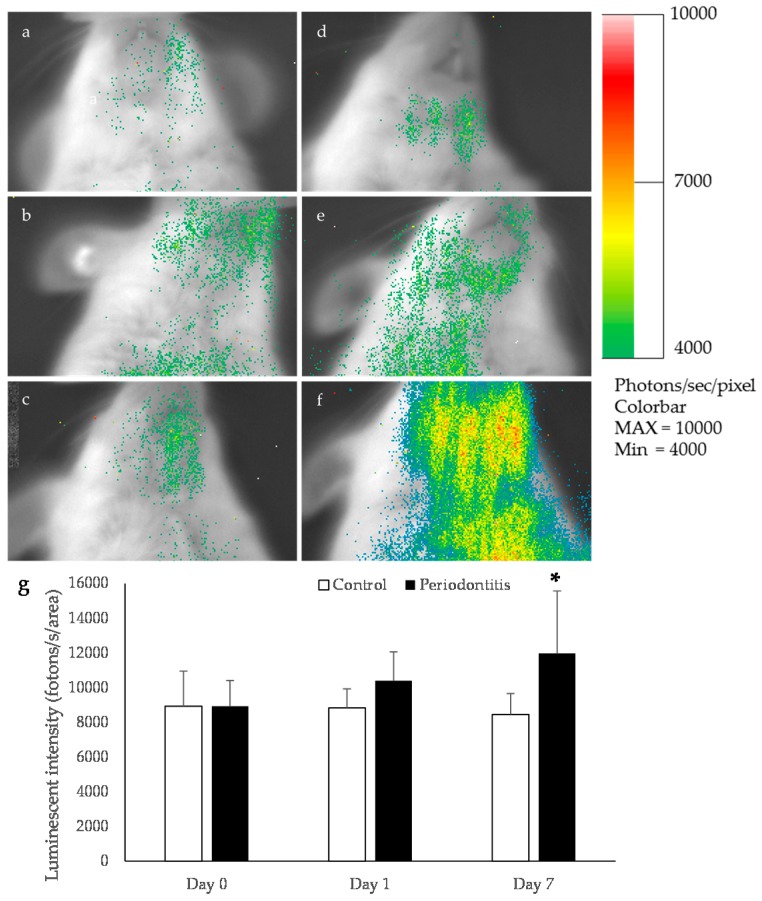
Bioluminescence imaging in oxidative stress detector-luciferase (OKD-LUC) mice. The color scale bar shows the photon counts. Representative photographs taken at baseline (**a**); the control group at day 1 (**b**) and day 7 (**c**); the periodontitis group just after ligation (**d**); and the periodontitis group at day 1 (**e**) and day 7 (**f**). In the periodontitis group, a high luminescence intensity was observed in the mandibular area at day 7 (**d**). This luminescence intensity was significantly higher in the periodontitis group than in the control group (* *p* < 0.05, *t*-test) (*n* = 6/group) (**g**).

A higher luminescence intensity was observed in the mandibular area of the periodontitis group than in the control group at day 7 (Figure 3). This intensity was significantly greater in the periodontitis group than in the control group (*p* < 0.05) (Figure 3).

In real-time polymerase chain reaction (PCR) analysis, the relative expression of HO-1 per glyceraldehyde-3-phosphate dehydrogenase (GAPDH) in the periodontitis group was 1.7 times higher than that of the control group.

**Figure 4:**
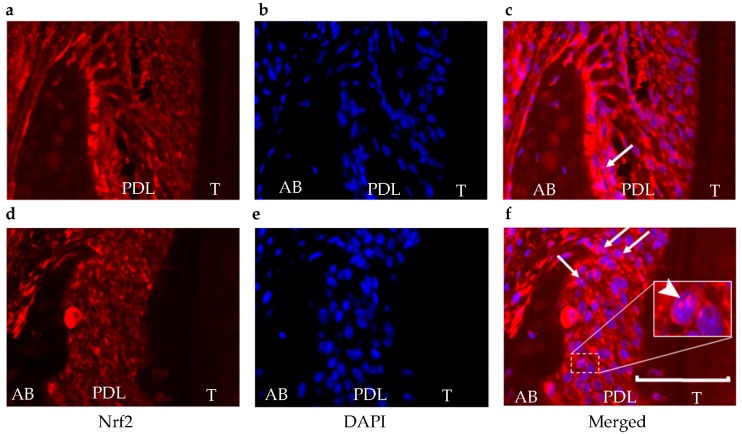
Translocation of Nrf2. For the Nrf2 nuclear translocation assay, the nuclear level of Nrf2 was determined using immunofluorescence staining (**red**) (**a**,**d**). The cell nuclei were visualized by DAPI staining (**blue**) (**b**,**e**). In the control group, Nrf2 expression was observed (**a**), but there was less translocation (**c**) than in the periodontitis group (**f**). Arrows show cells that are positive for both Nrf2 and DAPI in the nucleus. The arrowhead in white box shows a magnification of an area of cells with positive staining for both Nrf2 and DAPI in the nucleus. These double-positive cells mainly existed adjacent to the alveolar bone surface within the periodontal ligament. 4′,6-diamidino-2-phenylindole, DAPI; AB, alveolar bone; PDL, periodontal ligament; T, tooth. Scale bar = 50 μm.

Using an Nrf2 nuclear translocation assay, the nuclear level of Nrf2 was investigated using immunofluorescence staining to support the data of luminescence intensity. Nuclear Nrf2 was minimally expressed in the control group. It was observed more frequently in areas adjacent to the alveolar bone surface within the periodontal ligament in the periodontitis group (Figure 4).

**Figure 5:**
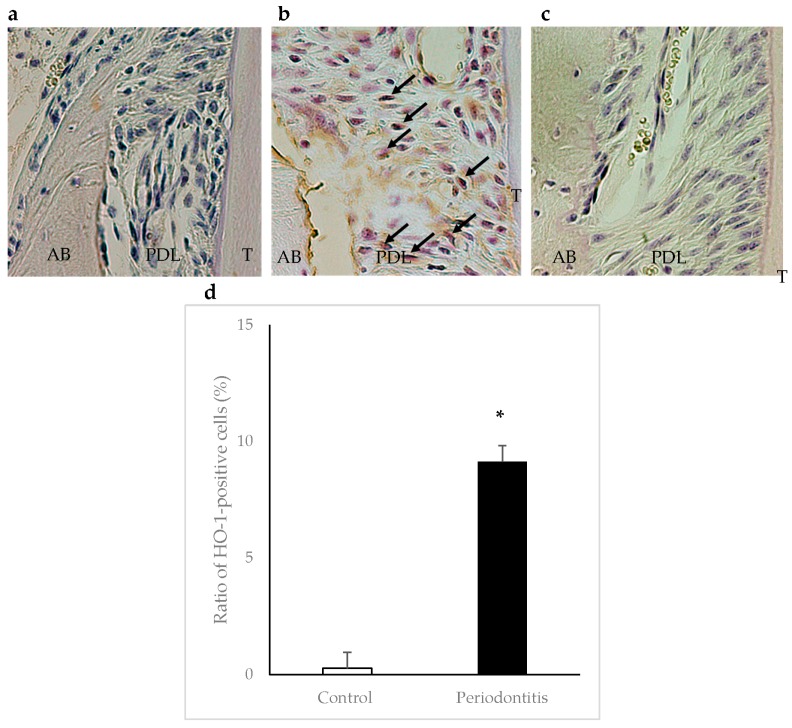
Immunohistochemical staining of heme oxygenase-1 (HO-1) in periodontal tissue at day 7. Arrows show HO-1-positive cells. A higher number of HO-1-positive cells was observed in areas adjacent to the alveolar bone surface within the periodontal ligament in the periodontitis group (**b**) than in the control group (**a**) (×400). Panel (**c**) shows a negative control. The ratio of HO-1-positive cells was significantly higher in the periodontitis group than in the control group (* *p* < 0.05, *t*-test) (*n* = 6/group) (**d**). AB, alveolar bone; PDL, periodontal ligament; T, tooth.

In the periodontitis group, HO-1-positive cells were observed in areas adjacent to the alveolar bone surface within the periodontal ligament at day 7. The ratio of HO-1-positive cells was significantly higher in the periodontitis group than in the control group (*p* < 0.05) (Figure 5).

**Figure 6:**
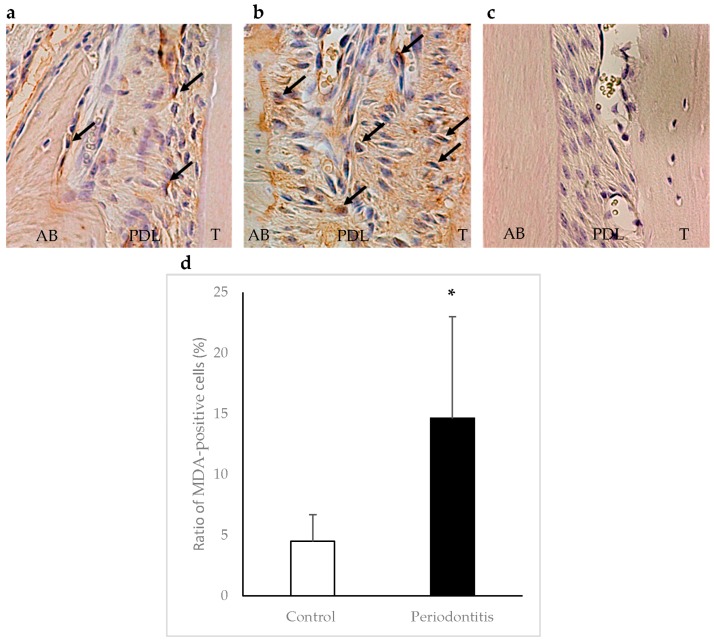
Immunohistochemical staining of malondialdehyde (MDA) in periodontal tissue at day 7. Arrows show MDA-positive cells. A higher number of MDA-positive cells was observed in areas adjacent to the alveolar bone surface within the periodontal ligament in the periodontitis group (**b**) than in the control group (**a**) (×400). Panel (**c**) shows a negative control. The ratio of MDA-positive cells was significantly higher in the periodontitisgroup than in the control group (* *p* < 0.01, *t*-test) (*n* = 6/group) (**d**). AB, alveolar bone; PDL, periodontal ligament; T, tooth.

In the periodontitis group, MDA-positive cells were observed in areas adjacent to the alveolar bone surface within the periodontal ligament at day 7. The ratio of MDA-positive cells was significantly higher in the periodontitis group than in the control group (*p* < 0.05) (Figure 6).

## 3. Discussion

Since the OKD-LUC mouse model has been developed and can be used for visualizing Nrf2 expression under oxidative stress [15], we used it to investigate the relationship between Nrf2 expression, oxidative stress, and periodontitis. In this study, luciferase activity and HO-1 expression were significantly greater in the periodontitis group than in the control group at day 7. Furthermore, in the immunofluorescent analysis, we confirmed that the nuclear translocation of Nrf2 occurred more frequently in the periodontitis group than in the control group. Nrf2 is the master regulator of antioxidant defense genes, and under oxidative stress, activated Nrf2 translocates into the nucleus to bind AREs [12]. Subsequently, Nrf2 activates antioxidant defense enzymes, including HO-1, to attenuate cellular oxidative stress [13]. Oxidative stress induced by periodontitis can also activate the Nrf2/antioxidant defense pathway [14,16]. Taken together, we interpreted that, under oxidative stress induced by experimental periodontitis, the Nrf2/antioxidant defense pathway was activated and could be visualized from the luciferase activity in the OKD-LUC model.

OKD48 transgenic mice can provide valuable information regarding oxidative status during development and under pathological conditions [15]. The OKD48 transgenic mice provide luminescent images and are useful for identifying living organs or cells under oxidative stress in vivo without fluorescent probes. In this study, the OKD48 transgenic mice actually permitted the visualization of oxidative stress due to periodontitis. We previously reported that oxidative stress due to periodontitis affects systemic conditions, and that antioxidants attenuated oxidative stress and inflammation [17,18,19]. Using this model, in the future, we may be able to visualize systemic oxidative stress by periodontitis in living animals, which may allow us to address various issues regarding oxidative stress in human chronic periodontitis and antioxidant development.

In periodontal tissue, both protein and gene expression levels of HO-1 were higher in the periodontitis group than in the control group. In rats, the gene expression level of HO-1 was significantly increased in a ligature-induce periodontitis group when compared to the control group in a three-week experiment [14]. Although the species and experimental period of that study were different from those of our study, the finding supports our results.

The level of MDA in periodontal tissue was significantly higher in the periodontitis group than in the control group. Our results indicated that ligature-induced periodontitis successfully increased oxidative stress in the periodontal tissue. When polyunsaturated lipids undergo a series of non-enzymatic reactions that produce a wide range of intermediate and end products due to ROS, MDA was the most specific molecule and the one most often used for the measurement of biological lipid oxidation [20]. MDA in serum, saliva, or gingival crevicular fluid is a marker of oxidative stress in human chronic periodontitis [21,22,23]. In experimental periodontitis, an increased MDA level was observed in the periodontal tissue [24]. For these reasons, we measured MDA in this study.

In our preliminary study, we confirmed the reproducibility of luciferase activity measurements, and the luciferase activity in the mandibular area peaked at one week after ligation and decreased after two weeks. The maximum change in alveolar bone loss was also observed within one week in the same model (data not shown). As such, we selected a one-week experimental period. However, the longer-term effects of the Nrf2/antioxidant pathway remain unknown, which was a limitation of this study.

Our study had other limitations. First, the bioluminescence signal was weak in our periodontitis model. The order of magnitude in the previous study was ×10^8^ [15], which was greater than in our study. We should contrive to enhance the signal to be able to detect minute changes in oxidative stress due to periodontitis. For example, the use of other types of periodontitis models that induce more oxidative stress [25] or luminescence proteins that have stronger light-emitting ability might allow us to overcome this problem. Second, we only focused on the Nrf2/HO-1 pathway as a major pathway of Nrf2/antioxidant defense. Further research on other pathways, such as NAD(P)H: quinine oxidoreductase 1, is required. Third, we just found the induction of the Nrf2-mediated anti-oxidative defense system, but did not find bone destruction as a result of the induction of the Nrf2-mediated anti-oxidative defense system. Considering a balance between the Nrf2-mediated anti-oxidative defense system and bone destruction, we need further experiments to explore the mechanism underling the balance.

## 4. Materials and Methods

### 4.1. Animals

All animal experiments were approved by the Animal Care and Use Committee of Okayama University (OKU-201323). Keap1-dependent Oxidative stress Detector, No-48-luciferase (OKD48) mice [15,26] were purchased from Trans Genic Inc., Ltd. (Kobe, Japan). Twelve male OKD48 mice (age, eight weeks) were housed in an air-conditioned room (23–25 °C) with a 12-h light-dark cycle. They had free access to standard chow (NMF; Oriental Yeast Co., Ltd., Osaka, Japan) and drinking water.

### 4.2. Experimental Design

The mice were randomly divided into two groups: a periodontitis group (*n* = 6) and a control group (*n* = 6). In the periodontitis group, a 5/0 cotton ligature was placed in the submarginal position on the mandibular first molars under intraperitoneal anesthesia (sodium pentobarbital at 0.5 mL/kg body weight) to induced periodontitis for seven days [25]. The mice in the control group received intraperitoneal anesthesia (sham treatment). We checked the body weight. There were no significant differences in body weight change between the periodontitis and control groups during the experimental period.

### 4.3. Bioluminescence Imaging

**Figure 7:**
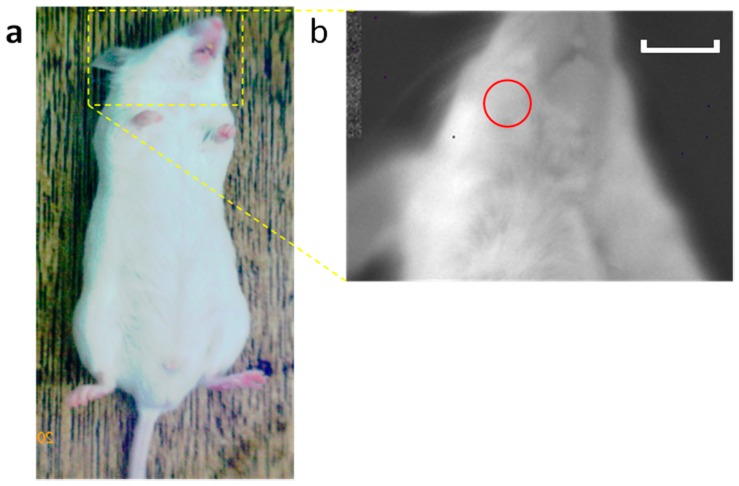
Scheme of bioluminescence imaging. Imaging of the head area was performed (**a**). The red circle shows the region of interest (ROI) (**b**). Luciferase activity was quantified from images displaying surface radiance using circular ROIs. Scale bar = 1 cm.

Bioluminescence imaging was performed as previously described [12]. Briefly, OKD48 mice were imaged using an imaging system (Lumazone^®^; Nippon Roper, Tokyo, Japan). Ten minutes before the imaging session, the mice received an intraperitoneal injection of a d-luciferin potassium salt solution (0.15 mg/g body weight) (Promega, Madison, WI, USA) and were anesthetized with an isoflurane/oxygen gas mix. Data were collected during a 5-min exposure. Imaging (one image per animal) was performed at days 0, 1, and 7. Luciferase activity was quantified from images displaying surface radiance using circular regions of interest (ROIs) [26] (60-pixel diameter circle in the mandibular area) (Figure 7) and then converted to the total flux of photons (photons/s) using software (MetaMorph, Nippon Roper).

### 4.4. Sampling

The mice were sacrificed under general anesthesia (diethyl ether) after seven days. For histological analysis, the right mandibular molar regions were resected en bloc from each mouse and fixed in 4% paraformaldehyde in 0.1 mol/L phosphate buffer (pH 7.4) for one day. Gingival biopsy samples of the left mandibular molar regions were homogenized using a frozen cell crusher (Microtec Co., Chiba, Japan) and used for real-time PCR.

### 4.5. Determination of Alveolar Bone Loss

After fixation, the mandible was stained with 1% methylene blue (Muto Pure Chemicals Co., Tokyo, Japan). Images of the mandible were captured using a Nikon digital camera D3200 (Nikon Instruments Inc., Tokyo, Japan). Horizontal bone loss, evaluated by the distance between the CEJ and the ABC along the long axis of the roots of the mandibular first molar at six sites corresponding to the mesio-lingual root, lingual furcation, disto-lingual root, mesio-buccal root, buccal furcation, and disto-buccal root, was measured. The values were calculated using mathematical morphology software (WinROOF; Mitani Co., Fukui, Japan).

### 4.6. Real-Time PCR Analysis

Total RNA was isolated from the gingival biopsy samples using Trizol reagent (Invitrogen, Carlsbad, CA, USA) according to the manufacturer’s instructions. Isolated RNA was quantified by measuring the absorbance at 260 nm, and purity was determined by the 260/280 nm absorbance ratio. Only samples with a ratio of >1.8 were used [27]. Total RNA was reverse-transcribed using AMV Reverse Transcriptase (Takara Bio Inc., Shiga, Japan) at 42 °C for 30 min. Real-time PCR was performed using SYBR Green Real-time PCR Master Mix (Toyobo, Osaka, Japan) in a real-time QPCR system (Agilent Technologies, Tokyo, Japan). The primer sequences for HO-1 [28] and GAPDH [29] are shown in Table 1. The amplification conditions were as follows: 42 cycles at 95 °C (5 s), 53 °C (30 s), and 72 °C (30 s) for GAPDH; 40 cycles at 95 °C (30 s), 60 °C (60 s), and 72 °C (60 s) for HO-1. The mRNA levels were calculated in terms of the relative copy number ratio of each mRNA to GAPDH for each sample.

### 4.7. Immunohistochemical Analysis

The right mandibular samples were decalcified with a 10% tetrasodium ethylenediaminetetraacetic acid aqueous solution (pH 7.4) for two weeks at 4 °C after fixation. The samples were embedded in paraffin following dehydration with ethanol (70%, 80%, 90%, and 100%) and immersion in xylene. The paraffin-embedded bucco-lingual 5-μm sections were stained for immunohistochemistry as described below.

To confirm Nrf2 translocation to the nucleus, the staining procedure included double-fluorescence staining of the slides. Antigen retrieval was performed using 10 mM citric acid at 98 °C for 30 min followed by incubation for 20 min at room temperature. A polyclonal antibody against Nrf2 (Santa Cruz Biotechnology Inc., Dallas, TX, USA) was diluted to 1:200 in phosphate-buffered saline [30]. Alexa Fluor 594-conjugated anti-rabbit IgG (1:250) (Thermo Fisher Scientific K.K., Kanagawa, Japan), which produces a red fluorescence with an excitation maximum at 561 nm and emission at about 594 nm, was used as a secondary antibody [31]. Afterwards, a mounting medium with 4′,6-diamidino-2-phenylindole (DAPI) (ImmunoSelect Antifading Mounting Medium; Dianova, Hamburg, Germany), which produces a blue fluorescence with an excitation maximum at 365 nm and emission at about 460 nm, was used to cover the slides [32].

Immunohistochemical staining for HO-1 and MDA was performed. Commercial kits (Nichirei Co., Tokyo, Japan) were used to determine the levels of HO-1 and MDA. Polyclonal antibodies against HO-1 (an indicator of anti-oxidation) (#ab85309; Abcam, Cambridge, MA, USA) and MDA (an indicator of oxidative damage) (#ab6463; Abcam) [33] were diluted to 1:400 and 1:500, respectively, in phosphate-buffered saline. For the negative controls, sections were processed as above, except the primary incubation was performed without the primary antibodies. The color was developed with 3-3′-diaminobenzidine tetrahydrochloride. Sections were counterstained with Mayer’s hematoxylin.

The numbers of HO-1-positive cells, MDA-positive cells, and total cells in standard areas (0.05 mm × 0.05 mm each) adjacent to the alveolar bone surface within the periodontal ligament (three serial areas from the top of the alveolar bone crest) were determined [4], and then the ratios of the positive cells were calculated. Three sections stained with HO-1 or MDA antibody from each animal were selected for the analyses.

### 4.8. Statistical Analysis

Power analysis and sample size were calculated using statistical software (SamplePower ver. 3.0; IBM, Tokyo, Japan) based on the results of bioluminescence intensity from a preliminary study. A sample size of six animals per group was required for the detection of significant differences in bioluminescent intensity with 85% power and a two-sided 5% significance level.

The data are presented as the mean ± standard deviation. The *t*-test was used for statistical comparisons of body weight, bioluminescent intensity, and the density of HO-1- or MDA-positive cells between the control and periodontitis groups. A statistical software (SPSS ver.21 for Windows; IBM) was used. A *p* < 0.05 was considered to be statistically significant.

## 5. Conclusions

Under oxidative stress induced by experimental periodontitis, the Nrf2/HO-1 pathway was activated and could be visualized from the luciferase activity in the OKD-LUC model. Thus, the OKD-LUC mouse model may be useful for exploring the mechanism underlying the relationship between the Nrf2/antioxidant defense pathway and periodontitis.

## Figures and Tables

**Table 1:** Primer sequences.

Gene	Primer Sequences (5′-3′)	Accession No.	Length (bp)
*HO-1*	F: CGT GCT CGA ATG AAC ACT CT	NM_010442.2	266
	R: GGA AGC TGA GAG TGA GGA CC		
*GAPDH*	F: TGT GAT GGG TGT GAA CCA CGA GAA	NM_001289726.1	130
	R: GAG CCC TTC CAC AAT GCC AAA GTT		
